# A Bioluminescence-Based Drug Screen Identifies Activities of Fexinidazole and Its Metabolites against *Helicobacter pylori*

**DOI:** 10.3390/antibiotics11111605

**Published:** 2022-11-11

**Authors:** Abdolhakim Mohamed, John N Chilingerian, Prerna Bali, Marygorret Obonyo, Anjan Debnath

**Affiliations:** 1 Department of Medicine, University of California San Diego, 9500 Gilman Drive, MC 0640, La Jolla, CA 92093-0640, USA; 2 Center for Discovery and Innovation in Parasitic Diseases, Skaggs School of Pharmacy and Pharmaceutical Sciences, University of California San Diego, 9500 Gilman Drive, MC 0657, La Jolla, CA 92093-0657, USA

**Keywords:** *Helicobacter pylori*, compound screen, fexinidazole, antibacterial

## Abstract

*Helicobacter pylori* is responsible for a wide range of gastric diseases, including gastric cancer and gastritis. With half of the world’s population infected by *H. pylori* and the current standard of care associated with suboptimal outcomes, a search for more effective drugs is critical. To facilitate drug screening for *H. pylori*, we developed a microtiter plate-based compound screening method that is faster and can screen multiple compounds. We identified activities of fexinidazole and its sulfoxide and sulfone metabolites against *H. pylori*. Both fexinidazole and its metabolites exhibited equipotency against SS1, 60190, and G27 strains, which were about 3–6-fold more potent than the currently used metronidazole. We also determined the minimal inhibitory concentration (MIC) of metronidazole, fexinidazole, and its metabolites against these strains by a traditional agar plate-based method. While MIC values of fexinidazole and metronidazole were similar against all the strains, both sulfoxide and sulfone showed lower MIC values than metronidazole against SS1 and 60190. Given the recent FDA approval of fexinidazole, our data on the in vitro antibacterial activities of fexinidazole and its metabolites support further evaluation of this drug with the goal of producing an alternative nitro-based antimicrobial with good safety profiles for the treatment of *H. pylori* infection.

## 1. Introduction

*Helicobacter pylori* is the most common bacterial pathogen in the world, infecting almost half of the world’s population [1]. It significantly impacts global health as the main cause of chronic gastritis, peptic ulcers, and gastric malignancy [1,2,3,4,5]. In addition, it has recently become clear that chronic infection with *H. pylori* can exacerbate other disease conditions, including self-limiting infections, stroke, and ischemic heart disease, due to *H. pylori*-induced host responses [6,7]. Eradication of *H. pylori* is mandatory in the prevention and management of these gastroduodenal diseases. Once detected, treatment is complicated and expensive, requiring combinations of antibiotics and proton pump inhibitors (PPIs) administered for extended periods [8,9,10]. Even with optimal management, treatment failures remain an increasingly serious problem [11,12,13]. Further, even after successful treatment, reinfection can occur, with rates as high as 30% per year [14,15,16]. Therefore, the discovery and development of new anti-*H. pylori* drugs with both superior therapeutic efficacy and negligible adverse effects are a critical unmet need to treat *H. pylori* infection.

Generally, screening of compounds against *H. pylori* involves investigating the minimal inhibitory concentration (MIC) of specific compounds through procedures such as serial broth dilutions or the Kirby–Bauer method [17]. However, *H. pylori* grows slowly in contrast to other commonly cultured bacteria, which can make traditional screening methods for *H. pylori* time-consuming and cost-inefficient. To overcome these drawbacks, recent studies have used high-throughput screening (HTS) technology that is fast, including the Fourier transform infrared (FTIR) spectroscopy [18,19]. In this study, we modeled a HTS assay designed to screen multiple compounds for another microaerophilic pathogen, *Entamoeba histolytica* [20], to screen different compounds for *H. pylori*. Our procedure involved using a modified broth dilution method that screened compounds in 96-well broth cultures of *H. pylori* using the commercially available BacTiter-Glo Microbial Cell Viability Assay (Promega) to determine the half-maximal effective concentration (EC_50_) of each compound. This assay measures ATP bioluminescence generated when luciferase catalyzes the transformation of luciferin into oxyluciferin, yielding PPi, AMP, and light in the presence of cellular ATP and oxygen. The whole organism screening formatted to 96-well microtiter plates represents a rapid, sensitive, and more efficient assay for screening multiple compounds to identify bactericidal compounds. The screen was validated with the currently used drug metronidazole. Since nitroimidazole scaffolds have an established history of anti-*H. pylori* activity, the use of nitroimidazole drugs already approved for human use opens the possibility to rapidly and cost-effectively repurpose drugs to treat *H. pylori* infection. We used our newly developed screening method to investigate the activity of a rediscovered nitroimidazole compound, fexinidazole, and its metabolites fexinidazole sulfoxide and fexinidazole sulfone. Fexinidazole is inexpensive and was recently approved by the U.S. Food and Drug administration (FDA) [21]. This is a promising alternative and safe nitro-based antimicrobial drug, considering *H. pylori* treatment failures due to an increase in resistance to the current metronidazole drug [22,23,24,25,26]. After confirming the activities of these compounds in a 96-well plate liquid culture, we determined the MIC of these compounds in a traditional agar plate-based method.

## 2. Results

### 2.1. Viability Assay for H. pylori

**Figure 1:**
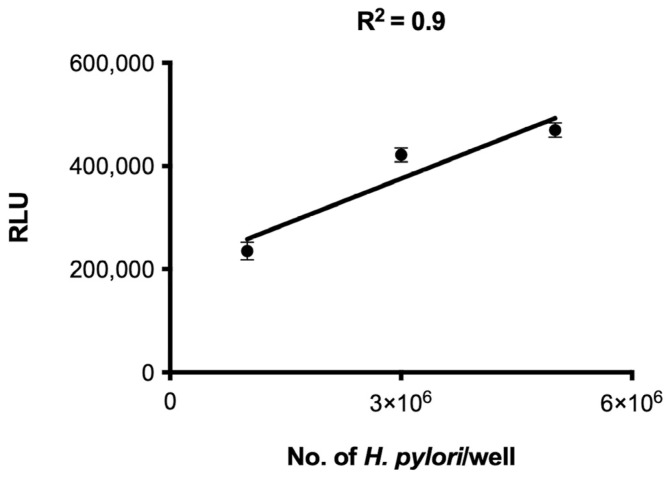
Correlation between *H. pylori* CFU and ATP bioluminescence in a 96-well microtiter plate. Different numbers of bacteria were seeded in a 96-well plate and ATP bioluminescence was measured after 24 h of incubation. Values plotted are the means and standard deviations of triplicate wells. The line represents a regression curve for the plotted data. RLU, relative light unit.

Screening methods on agar plates to assess the bactericidal activity of compounds against *H. pylori* are slow-paced, labor-intensive, and not amenable to high-throughput screening. To accelerate the identification of anti-*H. pylori* compounds, we miniaturized the assay that relies on the correlation of the *H. pylori* colony-forming unit (CFU) and the intracellular ATP generated by the organism. When different *H. pylori* CFU were seeded into 96-well microtiter plates, the luminescence generated from the bacteria demonstrated a strong linear correlation (R^2^ = 0.9) (Figure 1). A total of 3 × 10^6^ *H. pylori* CFU per well in a 96-well plate was used in subsequent BacTiter-Glo cell viability assays.

### 2.2. In Vitro Activity of Metronidazole, Fexinidazole, Fexinidazole Sulfoxide, and Fexinidazole Sulfone against Various H. pylori Strains

**Figure 2:**
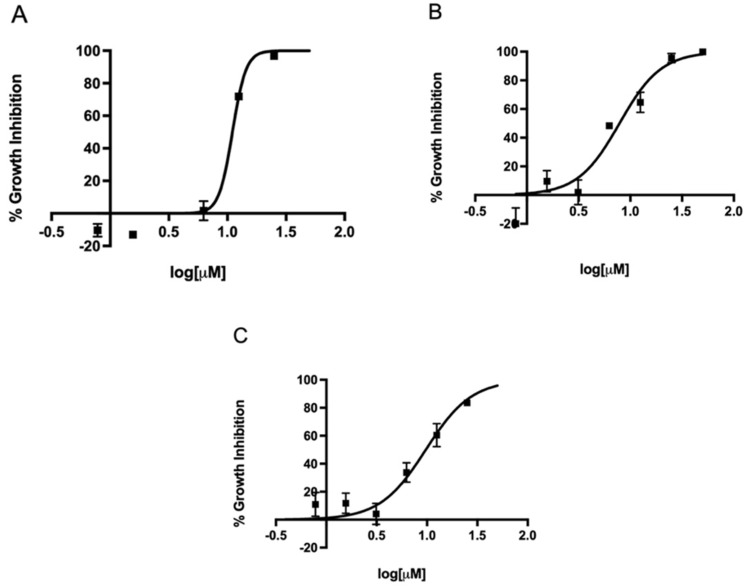
Concentration-dependent inhibition of growth of *H. pylori* by metronidazole. Strains SS1 (**A**), 60190 (**B**), and G27 (**C**) were treated with different concentrations of metronidazole for 24 h and EC_50_ curves were generated from mean values of percentage growth inhibition ± SEM of metronidazole against *H. pylori*.

**Figure 3:**
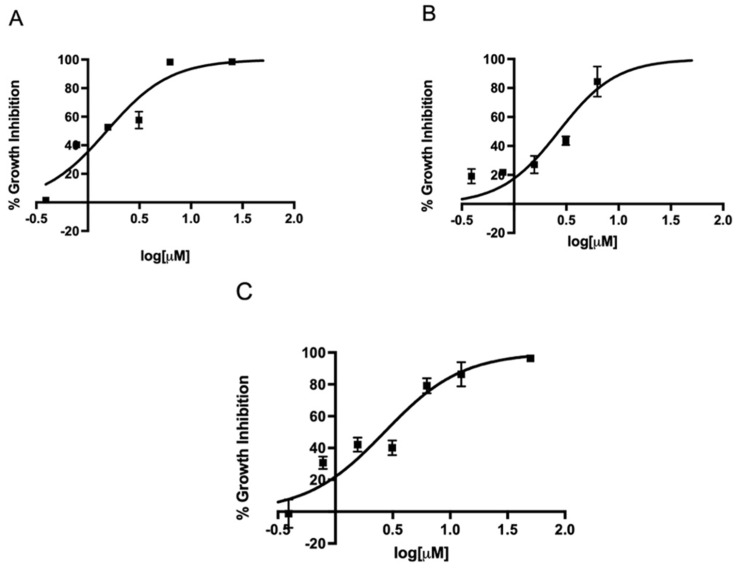
Concentration-dependent inhibition of growth of *H. pylori* by fexinidazole. Strains SS1 (**A**), 60190 (**B**), and G27 (**C**) were treated with different concentrations of fexinidazole for 24 h and EC_50_ curves were generated from mean values of percentage growth inhibition ± SEM of fexinidazole against *H. pylori*.

**Figure 4:**
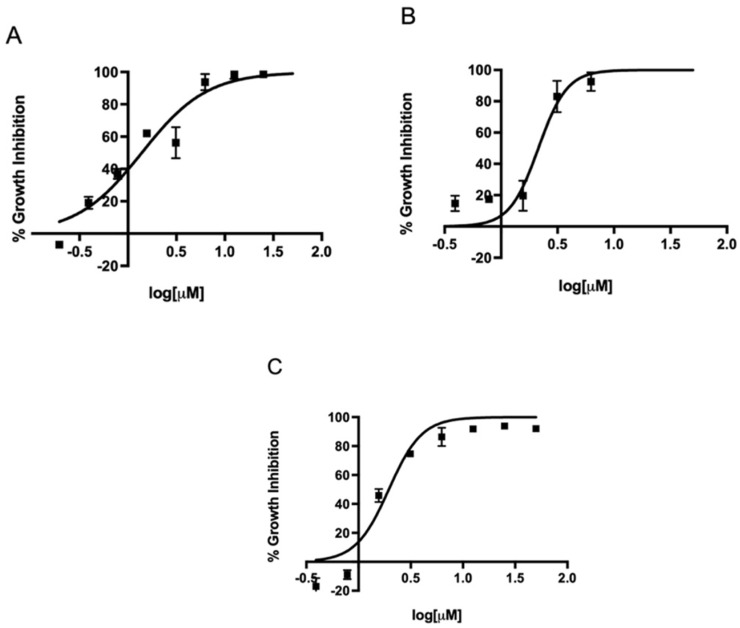
Concentration-dependent inhibition of growth of *H. pylori* by fexinidazole sulfoxide. Strains SS1 (**A**), 60190 (**B**), and G27 (**C**) were treated with different concentrations of fexinidazole sulfoxide for 24 h and EC_50_ curves were generated from mean values of percentage growth inhibition ± SEM of fexinidazole sulfoxide against *H. pylori*.

**Figure 5:**
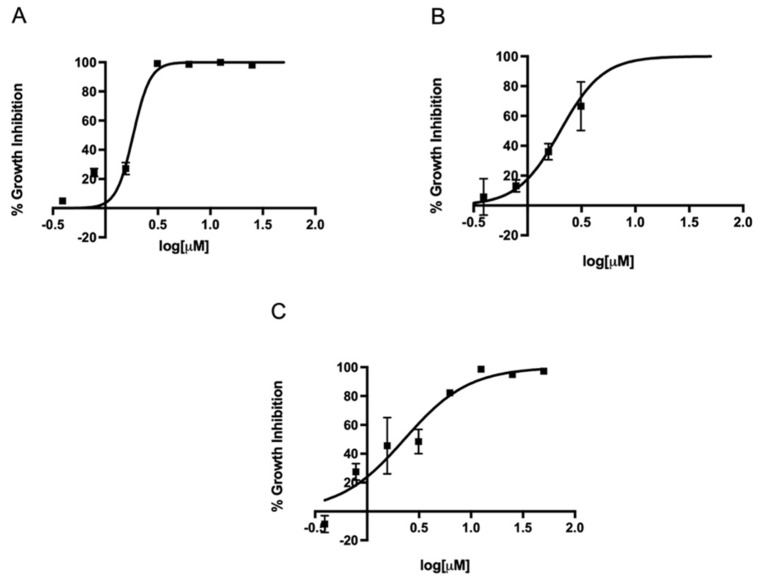
Concentration-dependent inhibition of growth of *H. pylori* by fexinidazole sulfone. Strains SS1 (**A**), 60190 (**B**), and G27 (**C**) were treated with different concentrations of fexinidazole sulfone for 24 h and EC_50_ curves were generated from mean values of percentage growth inhibition ± SEM of fexinidazole sulfone against *H. pylori*.

Since metronidazole is one of the antibiotics used in the standard triple therapy for *H. pylori* infection, we first determined the EC_50_ of metronidazole against three different strains in our microtiter plate-based assay. The EC_50_ of metronidazole against strains 60190, G27, and SS1 ranged from 7.8 to 11.1 µM (Figure 2). Once our assay could determine the EC_50_ of metronidazole on different strains, we expanded the assay to investigate the effect of the FDA-approved 5-nitroimidazole derivative fexinidazole and its metabolites fexinidazole sulfoxide and fexinidazole sulfone on these three strains. The parent drug fexinidazole and the metabolites exhibited about 2 µM EC_50_ against all three strains (Figure 3, Figure 4 and Figure 5). These growth inhibition experiments suggest that fexinidazole and the metabolites have broad activity against *H. pylori* strains, and they are about 4–6-fold more potent than metronidazole (Table 1). The screening of these compounds also generated a Z′ of 0.7–0.8.

### 2.3. MIC Determination of Metronidazole, Fexinidazole, Fexinidazole Sulfoxide, and Fexinidazole Sulfone against H. pylori Strains

**Figure 6:**
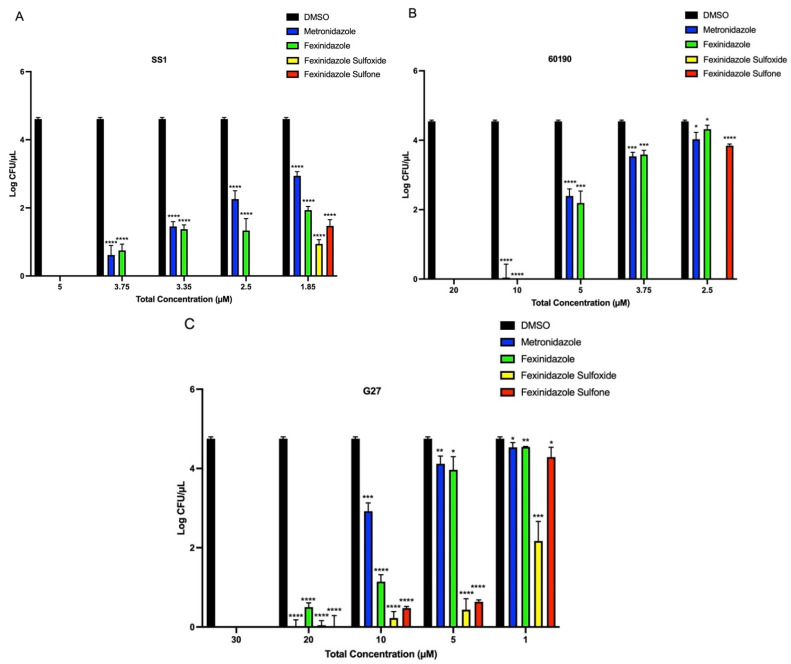
The MIC values of metronidazole, fexinidazole, fexinidazole sulfoxide, and fexinidazole sulfone against different strains of *H. pylori*. Strains SS1 (**A**), 60190 (**B**), and G27 (**C**) were treated with 1% DMSO or different concentrations of metronidazole, fexinidazole, fexinidazole sulfoxide, and fexinidazole sulfone for 24 h and colonies in agar plates were counted for bacterial growth. Values plotted are means and standard deviations from three different experiments. * *p* < 0.05, ** *p* < 0.01, *** *p* < 0.001, and **** *p* < 0.0001 by Student’s *t*-test compared to DMSO-treated *H. pylori*.

Considering the potency of fexinidazole and the metabolites on three strains of *H. pylori*, we also determined the MIC of these compounds in a traditional agar-based assay and compared it with the MIC of one of the standard antibiotics, metronidazole. While 24 h incubation of the SS1 strain with 5 µM of metronidazole or fexinidazole completely inhibited the growth of bacteria, both sulfoxide and sulfone metabolites exhibited an MIC of 2.5 µM (Figure 6A). For the strain 60190, both metronidazole and fexinidazole had similar MIC values of about 10 µM, whereas sulfoxide and sulfone metabolites demonstrated about 2.5- to 4-fold lower MIC values than fexinidazole, with sulfoxide having an MIC of 2.5 µM and sulfone having an MIC of 3.75 µM (Figure 6B). Metronidazole, fexinidazole, and the metabolites could not completely inhibit the growth of the G27 strain at lower concentrations and the MIC value of these compounds was 30 µM, which was much higher than the MIC values obtained with the other two strains (Figure 6C).

## 3. Discussion

The current worldwide standard treatment of *H. pylori* infection, termed standard triple therapy, consists of the administration of a proton pump inhibitor (PPI) and a combination of two antibiotics (clarithromycin plus amoxicillin or metronidazole) for at least seven days [8,9,10]. However, this therapy is associated with poor compliance of patients, side effects of the antibiotics, and high cost. Moreover, the increasing emergence of *H. pylori* strains resistant to some of the antibiotics has resulted in a progressive decline in recent years to unacceptable low eradication rates ranging from 60% to 75% [22,23,24,25,26,27,28]. It is now under discussion whether it is still ethical to continue the use of standard triple therapy in light of its declining efficacy [26]. To improve the eradication rates, alternative therapies such as quadruple and sequential treatment regimens have been suggested [26,27,29]. However, these therapies cannot address the problem of the growing trend in antimicrobial resistance of *H. pylori*. To combat these issues, new, effective, and safe drugs that are fast-acting, thereby reducing side effects, are urgently needed.

To accelerate the identification of new lead compounds for the treatment of *H. pylori* infection, we adapted a luciferase-based cell viability assay previously used to accurately identify potent compounds against other microaerophilic pathogens [20,30]. This simple luminescence-based assay is less labor-intensive and does not rely on extensive staining methods or colony counting. Our assay in a 96-well microtiter plate opens the possibility of developing a high-throughput screen for *H. pylori* by interfacing the assay with workstation-based automation. This will facilitate the screening of a large compound library and streamline the identification of lead compounds and subsequent determination of the structure–activity relationship in vitro.

Our assay was validated with metronidazole, one of the current drugs used in the treatment of *H. pylori* infection. Although metronidazole has been in clinical use for over 50 years, the expanded potential of metronidazole-based agents for microaerophilic pathogens has recently been demonstrated by others and our group [31,32,33,34,35]. Re-examination of ‘old’ nitroimidazoles is a valuable strategy in the development of new drugs for treatment of both parasitic and bacterial diseases. For example, a nitroimidazole drug, fexinidazole, was found highly effective and safe against sleeping sickness caused by *Trypanosoma brucei gambiense,* and it has received FDA approval for the treatment of human African trypanosomiasis [21] and was included in the WHO’s List of Essential Medicines. A detailed analysis of the genotoxic potential of fexinidazole was undertaken [36], and these studies suggested that fexinidazole may not be genotoxic. Previous studies showed that fexinidazole is rapidly metabolized in vivo through oxidation to at least two biologically active sulfoxide and sulfone metabolites and the blood concentrations of these metabolites exceed that of fexinidazole, suggesting that the metabolites are the therapeutically relevant species in vivo [36]. Free fractions of metabolites in human studies were about 60% and 43% for sulfoxide and sulfone, respectively, indicating that neither metabolite is highly protein-bound [37].

Considering an increased interest in identification of antibacterial nitro-heterocyclic compounds [31,35,38,39,40], we initiated investigations of fexinidazole and its two principal metabolites, fexinidazole sulfoxide and fexinidazole sulfone, against *H. pylori*. Moreover, the recent FDA approval of fexinidazole makes it a good candidate to repurpose for the treatment of *H. pylori* infection cost-effectively. The effect of fexinidazole and its metabolites on the activity of *H. pylori* was relatively fast, occurring within 24 h of incubation with about 2 µM of compounds, which reduced the growth of 50% of bacteria. The human clinical studies demonstrated that once-daily oral dosing of 1800 mg/day for 4 days led to plasma concentrations of sulfoxide and sulfone metabolites of 7.768 and 18.79 µg/mL, respectively, or 26.3 and 60.4 µM, respectively [37], which are about 13–30-fold more than the in vitro EC_50_ we identified against *H. pylori*. To our knowledge, this is the first reported evaluation of the antibacterial activity of fexinidazole and the metabolites against *H. pylori* strains.

Since the route of fexinidazole synthesis involves simple chemistry and shorter steps, the treatment cost with fexinidazole is expected to be relatively inexpensive. For the treatment of African sleeping sickness, it was calculated to be not more than USD 50 per treatment, or likely significantly less [36]. Considering the shorter treatment schedule than African sleeping sickness, the total cost for the treatment of *H. pylori* infection might be significantly less than the cost of treatment for African sleeping sickness. Future studies will determine the activity of fexinidazole and the metabolites on dormant forms and metronidazole-resistant *H. pylori* strains and evaluate the in vivo efficacy in a mouse model of *H. pylori* infection.

## 4. Materials and Methods

### 4.1. Chemicals and Reagents

White, solid-bottom tissue culture-treated 96-well microplates were purchased from Greiner Bio-One (Monroe, NC, USA). BacTiter-Glo Microbial Cell Viability Assay was purchased from Promega (Madison, WI, USA), dimethyl sulfoxide (DMSO) and metronidazole were purchased from Sigma-Aldrich (St. Louis, MO, USA), and fexinidazole, fexinidazole sulfoxide, and fexinidazole sulfone were provided by the Drugs for Neglected Diseases *initiative* (DND*i*) through Epichem Pty Ltd. (Bentley, WA, Australia).

### 4.2. H. pylori Bacterial Culture

*H. pylori* strains SS1, G27, and 60190 were used in this study. *H. pylori* SS1 is a mouse-adapted strain [41], and G27 (kind gift from Karen Guillemin, University of Oregon) [42] and 60190 [43] are clinical isolates. All strains were cultured on Columbia agar plates that were supplemented with 5% laked horse blood and 1% amphotericin B and incubated at 37 °C under microaerobic conditions (10% CO_2_, 85% N_2_, and 5% O_2_), as described in our previous studies [44,45,46]. The broth cultures were prepared by subculturing *H. pylori* in liquid media, brain heart infusion (BHI) supplemented with 5% fetal bovine serum (FBS), and then incubated for 24 h at 37 °C under microaerobic conditions on a reciprocal shaker [46].

### 4.3. H. pylori Strain Viability Assay

Broth cultures of *H. pylori* strain SS1 were centrifuged at 5000× *g* for 10 min to obtain a bacterial pellet and then resuspended in BHI supplemented with 5% FBS [13]. In a 96-well plate, each well was dispensed with 30 µL containing 1 × 10^6^, 3 × 10^6^, and 5 × 10^6^ *H. pylori* CFU and 170 µL of BHI supplemented with 5% FBS. The plates were incubated for 24 h at 37 °C under microaerophilic conditions on a reciprocal shaker. At the end of incubation, the plates were equilibrated to room temperature for 30 min and 100 µL of BacTiter-Glo Microbial Cell Viability Assay solution (Promega) was added to each well. The microplates were shaken on a microplate orbital shaker (VWR, Radnor, PA, USA) at 360 rpm for 10 min and the plates were incubated for an additional 10 min to stabilize the luminescent signal. The resulting ATP bioluminescence released by lysed *H. pylori* was measured by an EnVision 2104 Multilabel Reader (PerkinElmer, Waltham, MA, USA) at room temperature [47]. The growth assay was performed in triplicate in three independent experiments and the data were analyzed by GraphPad Prism 9.

### 4.4. In Vitro Activity of Metronidazole, Fexinidazole, Fexinidazole Sulfoxide, and Fexinidazole Sulfone against Various H. pylori Strains

Broth cultures of *H. pylori* strains SS1, G27, and 60190 were centrifuged at 5000× *g* for 10 min to obtain a bacterial pellet and then resuspended in BHI supplemented with 5% FBS [13]. In a 96-well plate, each well was dispensed with 30 µL containing 3 × 10^6^ *H. pylori* CFU, 169 µL of BHI supplemented with 5% FBS, and 1 µL of various concentrations (ranging from 0.0015 to 50 μM) of fexinidazole, fexinidazole sulfoxide, fexinidazole sulfone, or metronidazole. Here, 0.5% DMSO and 50 µM metronidazole served as negative and positive controls, respectively. The plates were incubated for 24 h at 37 °C under microaerophilic conditions on a reciprocal shaker. The growth inhibition assay was performed in triplicate in three independent experiments and the EC_50_ values of metronidazole, fexinidazole, and the metabolites were determined by the BacTiter-Glo Microbial Cell Viability Assay. The data were analyzed on GraphPad Prism 9 to determine EC_50_ values.

### 4.5. MIC Determination of Metronidazole, Fexinidazole, Fexinidazole Sulfoxide, and Fexinidazole Sulfone against Various H. pylori Strains

Broth cultures of *H. pylori* strains SS1, G27, and 60190 were centrifuged and resuspended as described previously. In a 96-well plate, each well was dispensed with 20 µL containing 1 × 10^6^ *H. pylori* CFU, 179 µL of BHI supplemented with 5% FBS, and 1 µL of various concentrations (ranging from 1.25 to 50 μM) of 1:1 serial dilution (starting at 30 or 50 μM) and one 1:2 serial dilution (starting at 2 μM) of either fexinidazole, fexinidazole sulfoxide, fexinidazole sulfone, or metronidazole. BHI medium with 1% DMSO served as a negative control. The plates were incubated for 24 h at 37 °C under microaerophilic conditions on a reciprocal shaker. Dilutions were performed for each individual well and then 10 µL of each diluted sample was inoculated onto Columbia agar plates supplemented with 5% laked horse blood and 1% amphotericin B [46]. The cultures were incubated overnight at 37 °C under microaerophilic conditions before colonies were counted.

## 5. Conclusions

In summary, we have demonstrated low micromolar EC_50_ of fexinidazole and the metabolites against three strains of *H. pylori*. Both fexinidazole and its metabolites were relatively fast-acting and exhibited activity within 24 h of incubation. The MIC values of fexinidazole and its metabolites were also in the low micromolar range against two strains of *H. pylori*. Considering the low cost, oral availability, and good safety profiles, fexinidazole warrants further investigation for repurposing for the treatment of *H. pylori* infection. We have also demonstrated that a bioluminescence-based assay is a faster and less labor-intensive method for screening and identifying drug activity against *H. pylori* compared to the labor-intensive serial broth dilution and colony-counting method.

## Figures and Tables

**Table 1:** EC_50_ values of fexinidazole, fexinidazole sulfoxide, fexinidazole sulfone, and metronidazole against *H. pylori*.

Compound	Strain	Mean (µM)	95% Lower CL (µM) ^1^	95% Upper CL (µM) ^1^
Fexinidazole	SS1	2	1.2	1.9
	60190	2.9	2.1	3.4
	G27	2.4	2	3.6
Fexinidazole sulfoxide	SS1	1.5	1.1	1.8
	60190	1.9	1.8	2.5
	G27	1.7	1.6	2.4
Fexinidazole sulfone	SS1	1.7	1.6	2.1
	60190	1.8	1.6	2.5
	G27	2.1	1.7	3.1
Metronidazole	SS1	11.1	6.1	19.9
	60190	7.8	6.1	10.1
	G27	9.3	6.1	15.4

^1^ CL, confidence limit.

## Data Availability

Data are contained within the article.
